# RETRACTION: Effect of Hydrocolloid Dressing on Pressure Ulcer in Patients with Non‐Invasive Positive Pressure Ventilation: A Meta‐Analysis

**DOI:** 10.1111/iwj.70150

**Published:** 2024-12-05

## Abstract

**RETRACTION:** LuoY.‐L. , LuoS.‐F. , LuoL. , OuM. , and TangM.‐L. , “Effect of Hydrocolloid Dressing on Pressure Ulcer in Patients with Non‐Invasive Positive Pressure Ventilation: A Meta‐Analysis,” International Wound Journal 21, no. 2 (2024): e14442, 10.1111/iwj.14442.PMC1082811937857589

The above article, published online on 19 October 2023, in Wiley Online Library (http://onlinelibrary.wiley.com/), has been retracted by agreement between the journal Editor in Chief, Professor Keith Harding; and John Wiley & Sons Ltd. Following an investigation by the publisher, all parties have concluded that this article was accepted solely on the basis of a compromised peer review process. In addition, the investigation found there was significant unattributed textual overlap between this article and a previously‐published article (Lin et al. 2023 [https://doi.org/10.1016/j.iccn.2023.103453]). The editors have therefore decided to retract the article. The authors did not respond to our notice regarding the retraction.

**RETRACTION:** Y.‐L. Luo , S.‐F. Luo , L. Luo , M. Ou , and M.‐L. Tang , “Effect of Hydrocolloid Dressing on Pressure Ulcer in Patients with Non‐Invasive Positive Pressure Ventilation: A Meta‐Analysis,” International Wound Journal 21, no. 2 (2024): e14442, 10.1111/iwj.14442.PMC1082811937857589

The above article, published online on 19 October 2023, in Wiley Online Library (http://onlinelibrary.wiley.com/), has been retracted by agreement between the journal Editor in Chief, Professor Keith Harding; and John Wiley & Sons Ltd. Following an investigation by the publisher, all parties have concluded that this article was accepted solely on the basis of a compromised peer review process. In addition, the investigation found there was significant unattributed textual overlap between this article and a previously‐published article (Lin et al. 2023 [https://doi.org/10.1016/j.iccn.2023.103453]). The editors have therefore decided to retract the article. The authors did not respond to our notice regarding the retraction.

